# Co-creating a decision-making framework for primary healthcare models in conflict-affected Cameroon and Nigeria

**DOI:** 10.1136/bmjgh-2025-019224

**Published:** 2026-03-26

**Authors:** Lundi-Anne Omam, Metuge Alain, Zara Wudiri, Chandini Aliyou Moustapha, Aisha Nuraini, Solomon Samuel Asimiya, Kelli N O’Laughlin, Roussel Ambebe, Jeremiah Alfred, Elizabeth Jarman, Steven Martin, Atabong Emmanuel Njingu, Iko Musa, Ful Morine Fuen, Loic Choupo, Lucky Andu, Simon Manuel, Olivia Acha-Morfaw, Chenwi Elvis Fuh, Tendongfor Nicholas, Mbui Idrissou, Hassan Mohammed, Tine Van Bortel, Rosalind Parkes-Ratanshi

**Affiliations:** 1 Public Health and Primary Care, University of Cambridge, Cambridge, UK; 2 Centre for Public Health, School of Medicine, Dentistry and Biomedical Sciences, Queen's University Belfast, Belfast, UK; 3 Health, Reach Out Cameroon, Buea, Cameroon; 4 Department of Community Medicine, University of Maiduguri, Maiduguri, Nigeria; 5 PhD Candidate, Florida International University, Miami, Florida, USA; 6 Action Against Hunger, Maiduguri, Nigeria; 7 Department of Nursing and Midwifery, National Open University Jalingo Nigeria, Maiduguri, Nigeria; 8 Department of Emergency Medicine, University of Washington, Seattle, Washington, USA; 9 Department of Public Health and Hygiene, University of Buea, Buea, Cameroon; 10 National Open University of Nigeria, Maiduguri, Nigeria; 11 De Montfort University, Leicester, UK; 12 International Medical Corps, Bamenda, Cameroon; 13 Cameroon Baptist Convention Health Service, Bamenda, Cameroon; 14 Department for the Disease, Epidemics and Pandemics Control, Ministry of Public Health, Etoudi, Cameroon; 15 Department of Public Health & Health Studies, University of Wolverhampton, Wolverhampton, UK; 16 Nigeria Centre for Disease Control and Prevention, Abuja, Nigeria; 17 Medicines for Humanity, Bamenda, Cameroon; 18 Community Health and Social Development for Cameroon - (COHESODEC), Bamenda, Cameroon; 19 Department of Primary Health Care, Ministry of Health Cameroon, Yaoundé, Cameroon; 20 Herwa Community Development Initiative, Maiduguri, Nigeria; 21 Insights and Real World Evaluations Group, Health Innovation East, Health Innovation Network, Cambridge, UK; 22 Center for Public Health, Queen’s University Belfast, Belfast, UK

**Keywords:** Delivery of Health Care, Global Health, Public Health, Health services research, Decision Making

## Abstract

**Purpose:**

Several models of care are used in conflict-affected settings; however, existing guidance on service delivery in humanitarian settings primarily focuses on improving the delivery of services. There remains a crucial gap in providing guidance in the selection and use of models of care while considering key aspects of quality of care.

**Methods:**

A co-creation approach was used involving various stakeholders to develop a framework and quality of care toolkit to support the selection of models of care used to deliver primary healthcare services in conflict-affected settings. A four-phase process was used. Findings from the first three phases will be published elsewhere. However, the four phases included: conducting a desk review and survey mapping models of care and humanitarian organisations, followed by in-depth interviews of organisations and focus group discussions with displaced populations. Further in-depth interviews were conducted exploring coverage and gaps in relation to seven domains of quality of care. Finally, two stakeholder workshops brought together humanitarian health experts and community representatives.

**Results:**

The two co-creation workshops with 60 humanitarian, Ministry of Health, academic institutions and community representatives were organised in Cameroon and Nigeria from 31 May to 14 June 2023. Key outputs from the Cameroon workshop were the development and consensus on the advantages and disadvantages of each model of care and the development of a collection of guiding questions to assist in model of care selection. A key output from the Nigerian workshop was the development of a quality-of-care checklist.

**Conclusions:**

This decision-making framework and quality toolkit can be used by programmers to guide model of care selection and use, while considering quality aspects. This decision-making framework and toolkit provide a structured and logical approach to model of care selection and can be refined and made accessible to others for widespread application.

**Trial registration number:**

NCT05279105.

**WHAT IS ALREADY KNOWN ON THIS TOPIC**

- Models of care for primary healthcare (PHC) delivery in conflict-affected settings are often determined by funders and organisational capacity rather than being tailored to the specific needs of affected populations.
- Co-creation approaches involving diverse stakeholders have been shown to improve intervention relevance, quality and sustainability in global health settings.
- However, reports of co-creation processes for PHC in conflict-affected regions, particularly in Cameroon and Nigeria, remain limited.

**WHAT THIS STUDY ADDS**

- A co-creation process involving stakeholders from humanitarian organisations, government, academia and affected communities successfully developed a decision-making framework and quality toolkit to guide the selection and implementation of models of care in conflict-affected settings.
- It offers methodological insights on co-designing tools and frameworks in complex environments, emphasising how collaborative approaches can address challenges unique to these contexts.

**HOW THIS STUDY MIGHT AFFECT RESEARCH, PRACTICE OR POLICY**

- The co-created decision-making framework and quality toolkit provide practical tools for humanitarian programme designers and policymakers to ensure context-specific, quality-focused PHC service delivery.
- By presenting practical steps and lessons learnt in co-creation, we aim to contribute to the growing body of evidence on participatory methodologies in global health.

## Introduction

 Co-creation is the collaboration of stakeholders, including community members, health professionals, policymakers and researchers, in the development of interventions, problem-solving and decision-making processes within public health initiatives.^1 2^ Co-creation models in public health within low- and middle-income countries generally engage end-users at different moments of co-creating the intervention, with some researchers combining both co-creation with community-based participatory research approaches.^3^ Co-creation is a method that fosters active participation and equal partnership, emphasising the integration of diverse knowledge sources.^1 4^ It aims to enhance intervention effectiveness by leveraging the collective expertise of stakeholders,^5^ ensuring relevance, feasibility and acceptability of interventions and improving their overall impact.^2^ Although co-creation is becoming more widespread in health, reports of the process are scarce, with the majority from the private sector or academic institutions in high-income countries. Public health interventions are predominantly developed using a top-down approach, characterised by having a large evidence base and based on behaviour change theories. By comparison, using end-users in the co-creation of public health interventions is thought to increase adherence and effectiveness due to empowering end-users, leading to increased end-user satisfaction, a higher quality of service provision and the sustainability and scalability of primary healthcare (PHC) services.^6^,^8^ In Africa, co-creation processes have been adopted to support awareness, preparedness and response actions for meningitis outbreaks.^9^ Elsewhere, examples of co-created complex interventions have been shown to help WASH (water, sanitation and hygiene) service delivery to meet the needs of residents in informal settlements in Nairobi.^10^ In Durban, South Africa, co-creation has been used in the development of innovative technologies aimed at creating sustainable and socially acceptable hand hygiene systems.^11^ In Kenya, co-creation and experimentation have been used around HIV care.^12^ Co-creation has also been used to address chronic disease management in rural Peru,^2^ and in Southern India, co-creation has been used to control and mitigate zoonotic disease.^13^ In the humanitarian sector, co-creation offers a framework, shared language and practical tools for examining and addressing power dynamics, fostering equitable partnerships, promoting mutual benefit and strengthening the capacities of local organisations and affected communities.^14^

For PHC services to provide the needed public health gains in conflict settings, services must be available, acceptable, affordable and of good quality to the people in need.^15^,^17^ Furthermore, for services to be equitable and accessible, the models of care employed by the PHC service providers should be immediate, integrated and longer-term and should incorporate needs assessments, effective supervision, institutional improvement, cost-efficient investment in terms of human resources, infrastructure and capacity building of the service providers.^18^ At present, several models of care, including mobile clinics, community-based interventions, health facilities, home visits, outreach and telemedicine are used in conflict-affected settings,^18^,^20^ yet there is limited guidance to orientate the choice of models of care to use in delivering PHC services in conflict-affected settings, with model of care commonly being determined by funders and/or organisational capacity^19 20^ rather than carefully considering the several factors which could offer a more tailored and contextually relevant approach to service delivery in conflict-affected settings. Service delivery is greatly influenced by the context in which services are delivered.^19^,^21^ More so, models of care used in conflict-affected settings also impact the quality of healthcare delivered.^22^ Thus, highlighting the need to guide and streamline the decision-making process in conflict-affected settings.

Since 2016 in Cameroon, there has been an armed conflict in the Northwest and Southwest (NWSW) regions of Cameroon owing to violent confrontations between the state military and non-state armed groups, creating recurrent cycles of internal displacement. This conflict has severely affected the needs and number of people affected. While displacement figures change continuously, the NWSW regions account for a substantial portion, ranging from 450 000 to 679 000 individuals, within the total displaced population of 977 000.^9^,^11^ Moreover, the crisis has resulted in the destruction of healthcare infrastructure, the departure of healthcare professionals and the loss of essential medical equipment. Across both regions of the NWSW, 253 healthcare facilities (37% of all health facilities) are currently non-operational.^23^,^26^ Moreover, the crisis led to the destruction of numerous villages, neglect of road maintenance and severe disruptions to transportation, making movement extremely difficult or even impossible in some areas. Health personnel and facilities became targets of violence, including kidnappings and arson, leading to widespread abandonment of health centres and a sharp decline in the health system’s functionality. In addition, access to certain hard-to-reach communities, often only accessible by water, became more costly and challenging. For instance, the cost of travel from Kumba to Ekondo-Titi in the South West Region rose from 3.99 GBP pre-conflict to 19.96 GBP during the crisis.^26^

Northeast Nigeria represents one of the largest geopolitical zones in the nation, with nearly one-third of Nigeria’s total surface area. The conflict in Northeast Nigeria (Boko Haram conflict), rooted in religious ideology, has become one of Africa’s most violent insurgencies, marked by bombings, mass shootings, targeted killings of civilians, suicide attacks and the destruction of schools and health facilities. These events significantly restricted population movement, increased transportation costs and contributed to severe food insecurity. In the context of the ongoing conflict, the states of Borno, Adamawa and Yobe have been principally affected since 2002,^27^,^31^ with full-scale humanitarian interventions since 2016; however, approximately 12 million people who are living outside of government-controlled areas are cut-off from humanitarian aid completely, while others experience many barriers to lifesaving humanitarian aid, of which healthcare makes up the second largest sector.^32^ These conflicts directly impact how PHC services are delivered using different models of care.

Together, these factors have had profound negative impacts on the health of people living in Northeast Nigeria. There is sparse evidence to guide the selection and design of PHC services that improve and maintain quality care in humanitarian settings, like those of the NWSW regions of Cameroon and Northeast Nigeria. Various models of PHC are used in these settings^18^,^35^ with little guidance or consideration when selecting a particular model of care. Thus, necessitating the development of a framework and quality of care toolkit to support PHC selection and service delivery is crucial. To develop this framework and toolkit, a co-creation approach was used involving various stakeholders, including representatives from affected communities (internally displaced persons). The co-creation process aimed first to co-develop a decision-making framework for PHC models adapted for the conflict-affected settings of Cameroon and Nigeria. The second aim of this co-creation process was to develop a toolkit with a pragmatic set of quality considerations during the delivery of PHC services. In this paper, we share the participatory methodology used, the applicability of the framework and toolkit, lessons learnt and recommendations for future co-creation initiatives.

## Methodology

### Study design

**Figure 1:**
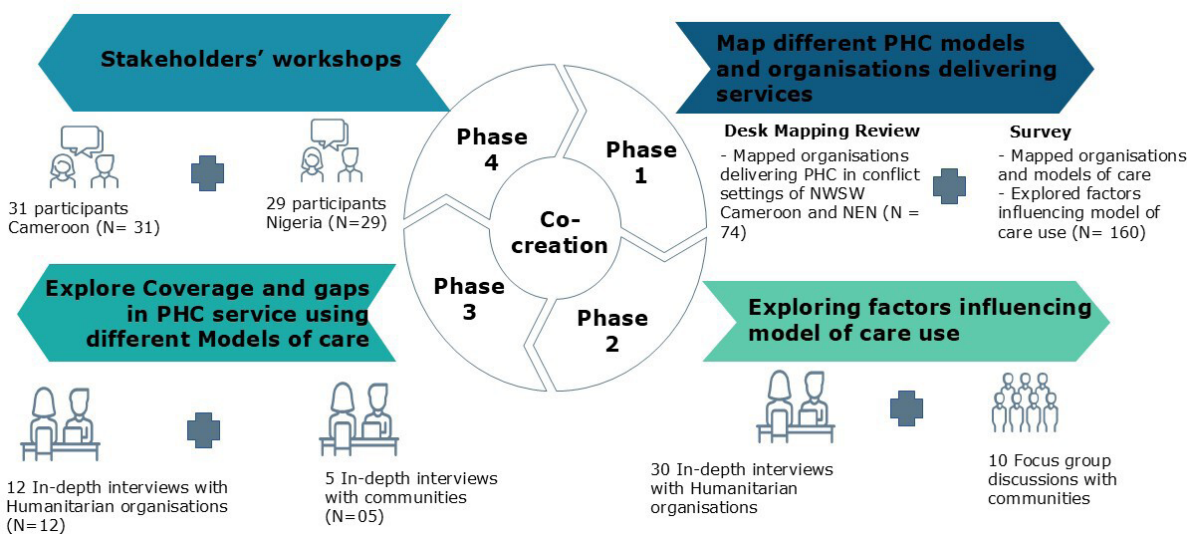
Illustration of action research process used in the co-creation process. NEN, Northeast Nigeria; NWSW, Northwest and Southwest; PHC, primary healthcare.

This decision-making framework and quality toolkit were developed using an action research approach, widely used in healthcare to study professional practice and patients’ experiences.^11^,^13^ A four-phase process was used with a wide and diverse range of humanitarian health experts and internally displaced persons (IDPs) to reach consensus (figure 1). In phase 1, a desk review and survey mapping the different models of care and humanitarian organisations in NWSW regions of Cameroon and North East Nigeria was conducted.^32^ In phase 2, in-depth interviews (IDIs) of organisations and focus group discussions (FGD) with displaced populations on factors influencing the model of care selection, including experiences on models of care selection. In phase 3, IDIs were conducted with humanitarian organisations and displaced populations, exploring coverage and gaps in relation to seven domains of quality of care.^7^ In phase 4, two stakeholder workshops brought together a diverse and multidisciplinary group of humanitarian health experts and community representatives. The first three phases, desk review and mapping, qualitative inquiry on the model of care selection and exploration of quality of care, were designed to build a strong empirical and experiential foundation. The fourth phase, stakeholder workshops, served to validate findings and co-develop practical tools with those directly involved in service delivery and affected by it. In this paper, we focus on the process and learnings from the fourth phase, the stakeholder workshops. Results and methods of phases 1 through 3 have been published or are being finalised for publication.^7^ The protocol for our study was registered on www.clinicalTrials.gov on 17 February 2022 with ID number NCT05279105. The co-creation process discussions were facilitated by a team of four researchers (three senior researchers and one junior researcher with extensive humanitarian experience), and two experienced humanitarian professionals.

### Participants

Two 3-day co-creation workshops were conducted in Maiduguri, Borno State, Nigeria and Limbe, Southwest Cameroon, bringing together a multi-sectoral group of stakeholders to finalise the process of co-creating a framework to inform decision-making on models of care use to deliver PHC in conflict-affected settings. The workshop in Cameroon was in-person only, while that of Nigeria was hybrid (in-person and online) due to security and travel restrictions for some facilitators of the workshop. Virtual co-creation workshops have previously been reported by Benson and coauthors.^36^ While the in-person workshop in Cameroon had all facilitators (research team) and participants physically present in the same venue, the hybrid workshop in Nigeria involved two facilitators (NBL-AO and RP-R) participating virtually via Microsoft Teams. Their presentations were delivered online, and interaction with participants during and after the presentations was facilitated by research team members (MA, ZW, MH, NN) who were physically present on-site. The presence of the aforementioned research team members in Nigeria (MA, ZW, MH, NN) ensured the quality of discussions, as the same team had also co-facilitated the workshop in Cameroon. Moreover, participant engagement in Nigeria mirrored that of the Cameroon workshop, given that the same co-facilitators led discussions in both settings.

Participants for both workshops were invited by email and included professionals from local and international non-governmental organisations (NGOs), United Nations (UN) agencies engaged in humanitarian response, government representatives from the Cameroon, Borno, Yobe and Adamawa State Ministries of Health, academics from local universities community leaders and IDPs residing in camps/settlements.^32^ Participants were identified with support from the health cluster leads who recommended participants from the database of humanitarian organisations working in both country contexts. Workshop participant details can be seen in table 1. Organisations which took part in the workshop focused on the following sectors: health, nutrition, food security, WASH and shelter. Government participants offered an in-depth understanding of the public sector of the primary care system and ongoing policy reconstruction efforts. Community leaders and IDPs imparted perspectives from affected communities across the conflict-affected countries. Academicians contributed critical reflections of the results from the survey, FGDs and IDIs, twice per day to corroborate findings with discussions happening in the workshop. NGO representatives shared crucial on-the-ground insights based on their experience selecting which model of care to use in providing PHC services and interventions in the NWSW regions of Cameroon and Northeast Nigeria’s complex emergency settings. IDP perspective provided insights to community involvement in programme design, exploring the involvement of communities in humanitarian programming and whether the model of care selection took into consideration their needs, realities and challenges in accessing healthcare. Inputs from IDPs were given equal consideration during the workshop. All participants were reimbursed for transportation fees and hotel accommodation (for participants not resident in the location of workshop) during the days of the work.

**Table 1:** Co-creation workshop participants per country

	Cameroon	Nigeria
United Nations	3	5
International non-governmental organisation	5	5
National non-governmental organisation	10	6
Community-based organisations	1	1
Faith-based organisations	2	0
Academic institution	4	2
Community leader	1	3
State ministry of health	–	2
Ministry of public health	3	–
Internally displaced person	2	3
Primary care agency	NA	2
**Total**	**31**	**29**

### Co-creation method (process)

Three workbooks (table 2) to orientate discussions and group works during the workshops were created using data obtained from the desk review, survey, IDIs and FGDs conducted with staff of humanitarian organisations and IDPs.^32^ Results from the aforementioned studies were presented during the workshops. Through facilitated discussion and group work, participants leveraged their multidisciplinary expertise and array of experiences in conflict-affected environments to co-create a contextualised framework intended to guide the selection and implementation of appropriate models of care for delivering comprehensive PHC services to crisis-impacted populations. Additionally, the workshop supported collaborative development of a toolkit expected to enhance coordination, efficiency and sustainability of efforts to rebuild equitable, quality PHC capacity in NWSW Cameroon and Northeast Nigeria. The quality considerations of this toolkit are based on the Global Health Cluster Quality of Care in Humanitarian Settings guidelines and WHO’s seven domains of quality of care in conflict-affected settings.^37^ Moreover, during the workshops, discussions were carried out in an iterative manner within groups who later presented what they agreed on to all the participants who could either disagree, modify or reject any point which was not applicable. Both workshops were conducted in English.

**Table 2:** Overview of workbooks, discussion points and group work tasks

Workbook	Focus and discussion points	Group work tasks
Workbook I	Presentation of results of the systematic review Discuss the general considerations needed for each model of care and reflect on the strengths and weaknesses	Reflect on other general considerations for service delivery for each category below. Suggest questions to inform the decision-making process for models of care Groups to reflect and suggest at least five considerations and recommendations
Workbook II	Presentation of survey and qualitative study on factors influencing use of models of care Reflect on the considerations, advantages and disadvantages of each model of care developed in workbook I The key reflective question for the group work: ‘Is there anything related to the given theme (eg, barriers to care) which will make this model of care not appropriate for a humanitarian context? If yes, then for each of the circumstances, write out what the most appropriate model might be, what other models could be considered, and which other model will be just as bad	Using the questions drafted in workbook II (checklist for decision making for models of care) and advantages, disadvantages in Workbook I, reflect/suggest considerations for ‘options’, recommendations, and notes to help guide the decision-making process on which model of care could be recommended following key considerations Each group to reflect and suggest at least five considerations and recommendations for their group

For both workshops, the first group work (workbook I) consisted of reviewing the considerations, advantages and disadvantages of each model of care developed from the studies conducted (systematic review, survey, IDIs and FGD). The models of care were health facilities, mobile clinics, community-based interventions, home visits, outreach, telemedicine, ambulances and hybrid models.^7^ Each group then presented to all participants for open discussion and validation of the work done. Validation was done by all participants agreeing on each point presented by the group who presented their work. Following this, participants then reflected and suggested guiding questions to assist humanitarian programme designers on the model of care selection (workbook II). Groups were given subthemes on which to focus. These subthemes included barriers, communities, healthcare financing, organisational capacity and humanitarian coordination. The groups were instructed to generate questions that led towards reflections of a preferred model or warned against a least preferred model in each situation. Each group had to test the questions for each model of care against the theme provided to them (online supplemental appendix 1). If the questions made sense to the group, they were retained and presented to all participants during plenary discussions.

The subthemes and codes that emerged from the qualitative interviews and the seven quality domains from the WHO quality of care publication for conflict-affected settings^37^ were used to develop an adapted toolkit for quality of care using the different models and per quality domain. The toolkit checklist was generated by the participants of the workshop. The subthemes and codes from the qualitative interviews provided guidance from which the checklist was developed during the workshop. The consolidated checklist of models of care and quality of care was then validated by workshop participants and the research team.

### Consensus and validation

The diverse representatives at the co-creation workshop achieved consensus on the need for a context-specific framework to guide coordinated efforts to rebuild quality, sustainable PHC capacity meeting crisis-affected communities’ needs in Northwest Southwest Cameroon and Northeast Nigeria. Consensus was reached during the workshops by all participants openly agreeing/validating all workshop outputs on the third day of each workshop.

Developing a practical guidance tool for selecting and implementing appropriate care models aligned with humanitarian principles through an inclusive process of co-creation as a priority objective. Despite recognising complexities in delivering healthcare amid emergencies, participants resolved that collaborative engagement during the model of care selection could support rebuilding equitable access to essential health services for vulnerable populations in conflict-affected settings.

### Patient and public involvement

Participants for both workshops included professionals from local and international NGOs, UN agencies engaged in humanitarian response, government representatives from the Cameroon, Borno, Yobe and Adamawa State Ministries of Health, academics from local universities community leaders and IDPs residing in camps/settlements.

## Results

### Cameroon workshop output

Following an extensive systematic review and the research done in Cameroon and Nigeria, seven models of care had been identified before the co-creation workshops. The first workshop was organised in Cameroon from 31 May to 2 June 2023 with 31 participants (10 women and 21 men). The workshop group works were centred around the eight identified models of care (online supplemental appendix 1). Key outputs from the workshop in Cameroon were the development and consensus on the key considerations for selecting each model, the advantages and disadvantages of using each model of care, along with the creation of a set of guiding questions to assist humanitarian programme designers in selecting appropriate models of care. Initial reflections on quality-of-care considerations for models of case use were garnered in Cameroon.

### Nigeria workshop output

The Nigeria workshop brought together 29 participants in the workshop, 25 men and four women, and took place from 12 to 14 June 2023. Most of the participants were from UN agencies (n=5), international NGOs (n=6) and local NGOs (n=6), as shown in table 1. The second workshop took place in Nigeria, Borno state from 12 to 14 June. The aim of the workshop was to validate the outputs (workbooks I and II) from the initial co-creation workshop in Cameroon (table 2). The validation process aimed to identify gaps and enhance the tools based on lessons learnt from the humanitarian response in Nigeria, specifically the Northeast. During group sessions focusing on workbook I (table 2), which dealt with models of care and their respective advantages and disadvantages. Participants in the Nigerian workshop identified the rapid response model (RRM) as a model of care for a humanitarian setting^26^ that had been used in Nigeria. The definition adapted for this RRM of care was described as ‘A model to reach inaccessible communities with sudden displacement requiring multisectoral emergency assistance’. The RRM differs from other models of care in that it provides not only health services to conflict-affected communities but also a multisectoral response that addresses other social needs. This often includes the provision of nutrition support, WASH assistance, household relief items and primary education, within 72 hours following the sudden displacement of vulnerable populations. It is similar to the mobile clinic model in that it uses an ambulatory approach to providing care. However, it differs in that it is typically activated within 72 hours following the sudden displacement of a vulnerable population and delivers a multisectoral response, unlike the mobile clinic, which provides only health services. Discussions on the RRM were not included in the Cameroon workshop because it was thought of as another form of mobile clinic. However, during the workshop in Nigeria, participants did not agree with this view and requested that it be regarded as a model in its own right.

The advantages and disadvantages of this model were identified. Advantages include its ability to serve a class of people under special needs, it improves access to services, reduces the course of transfer of cases, facilitates referral services, it is a holistic approach combining many interventions, capacitation of the local community, it reaches the most inaccessible places. However, disadvantages noted were its high cost, time-consuming and it can only be used in a short time to respond to the immediate needs of the affected communities.

Another key output from the Nigerian workshop was the development of a quality-of-care checklist. Participants in the Nigerian workshop recommended restructuring certain questions and incorporating additional queries before the final validation of the workbook III (table 1). The quality-of-care assessment tool also had minor modifications during the group session and was validated subsequent to the amendments. Some of the minor corrections included can be seen in table 3. However, the Nigerian co-creation workshop developed a quality-of-care evaluation tool that was an improved version of the work done during the Cameroon workshop. This quality-of-care evaluation tool can be used by humanitarian programmers to assess the quality of care for each model of PHC healthcare delivery during programme or project interventions in humanitarian/conflict settings, as outlined in table 3.

**Table 3:** Cross-cutting differences and similarities between Cameroon and Nigeria workshops

Workbook	Cameroon workshop	Nigeria workshop
Workbook I	The workshop in Cameroon focused on discussing around models of care, that is, health facilities, mobile clinics, community-based interventions, home visits, outreach and telemedicine, ambulances and hybrid models	The Nigeria workshop reviewed the initial outputs from the Cameroon workshop based on the eight models of care and suggested an additional model of care called ‘Rapid Response’ to the nine considered for inclusion in Nigeria
	Elaborated on the strengths and the weaknesses of each of the models of care	The participants agreed that community-based participatory approaches leveraging capabilities of both international partners and national health systems represent the most effective way forward
Workbook II	New barriers not previously identified in the studies conducted (systematic review, survey, qualitative study) were identified	After reviewing the barriers arrived at the Cameroon workshop, no additional barriers were identified by the Nigeria workshop
	Identified the key considerations humanitarian organisations should reflect on that could affect implementation of each model of care	The workshop achieved consensus on the need for a context-specific framework to guide coordinated efforts to rebuild quality, sustainable primary healthcare capacity meeting crisis-affected communities’ needs in Northeast Nigeria and Cameroon
	Developed guiding questions which, if answered, will lead humanitarian programme designers and managers to the most likely or most appropriate model of care to use Developed questions that lead towards a preferred model or warn against a less preferred model in each situation	Developed a practical guidance tool for selecting and implementing appropriate care models aligned with humanitarian principles through an inclusive process
Workbook III	Developed initial toolkit for quality-of-care assessment based on the quality-of-care domains. Toolkit to be used to assess programme quality prior to implementation of a particular model	Validated the first quality-of-care assessment toolkit for programmes developed during the Cameroon workshop Developed a second quality-of-care toolkit per model of care

Modifications made during the Nigerian workshop of work done in the Cameroon workshop can be seen in table 3.

## Discussion

To our knowledge, this is the first study to use a co-creation approach in developing a decision-making framework to guide model of care selection in conflict-affected settings of Cameroon and Nigeria. In Nigeria, co-creation of evidence was adopted between political decision-makers, health policymakers and academics during COVID-19^38^ and for delivering adolescent sexual and reproductive health interventions.^39^ Conversely, little work to our knowledge has been undertaken using co-creation or co-production methods in Cameroon.

After participating in two workshops informed by results from a mixed-methods study conducted in the same settings, a decision-making framework and quality toolkit was developed. This decision-making framework and toolkit appear to provide a structured and logical approach to model of care use. This framework provides an easy-to-use tool for organisations in conflict-affected settings to respond to critical questions when choosing a model of care in a predictable and reproducible manner, based on the local realities. The tool has the potential to be used by humanitarian organisations to guide the selection of models of care for conflict-affected settings. While there exist resources and guidelines in the humanitarian sector providing valuable orientation on improving service delivery to vertical programmes,^18^ the decision-making framework and quality toolkit provide orientation to service delivery through carefully selecting models to deliver a comprehensive set of services to communities. Moreover, the tool integrates elements of quality of care, general considerations and advantages and disadvantages of different models of care in the selection process of the most appropriate model of care. The tool can be integrated into an online platform where programme managers can easily fill in critical information about their context to get recommendations on the best model of care for their context. The co-creation approach we used in developing our decision-making framework and quality toolkit agrees with previously published co-creation approaches in conflict settings to equalise power dynamics within research partnerships,^40^ determine research priorities that are culturally appropriate and adapted to local settings^41^ and improve the quality and acceptability of decision-making^13^ and social accountability.^42^

Important aspects which may contribute to the success of future co-creation processes include the involvement of a wide range of stakeholders, including community members, health professionals, policymakers, researchers and NGOs. Future co-creation processes should prioritise inclusivity to ensure a comprehensive understanding of the healthcare challenges and potential solutions. Also, recognising the importance of adapting co-creation processes to the specific context is critical for future co-creation initiatives. More so, the validation of outputs and tools developed in co-creation workshops is crucial. Our workshops included a validation process where all group work was validated twice at plenary. This iterative approach ensures that the resulting frameworks and tools are practical and relevant.

### Limitations

This co-creation process had some limitations. First, the process of purposeful selection of stakeholders for the co-creation workshop may have caused bias. Despite the efforts to include a diverse range of participants, there might be a risk that certain voices or perspectives may have been over-represented or under-represented. This could affect the generalisability of the findings, as the resulting framework may be more reflective of the experiences and priorities of the selected participants rather than the wider range of stakeholders involved in similar settings. However, considering that the discussions and reflections were guided by findings from the systematic review, survey and qualitative inquiry and coupled with the inclusion of participants from diverse organisational types, geographic regions and community backgrounds, the relevance of the framework is strengthened, making it a valuable starting point for adaptation and application in other conflict-affected settings.

Also, organising co-creation workshops is resource-intensive and time-consuming. Gathering diverse stakeholders, conducting surveys, interviews and workshops requires significant financial and human resources. In many humanitarian settings, resources are limited, and time is of the essence, as reported by Leresche *et al*^43^ and Beran *et al*^44^ who recognised time and investments as key challenges for co-creation processes in sustaining effective operational research and humanitarian operations in conflict settings. Alternative methods such as Delphi panels, virtual consultations or structured stakeholder interviews could have been used to reduce resource demands.^45^ While these approaches may be cost-efficient, they often lack the depth of interaction, trust-building and real-time negotiation that in-person co-creation enables. Therefore, implementing such workshops on a broader scale may be challenging due to resource and time constraints.

Finally, the co-creation workshop primarily focused on developing a framework and toolkit for models of care selection and quality considerations for healthcare delivery in conflict-affected settings. While this is essential, it may not address all aspects of healthcare delivery in such complex and challenging operating environments. This could limit the framework and toolkit’s ability to comprehensively address the complex healthcare challenges in these conflict-affected countries. This work was specifically designed for the conflict-affected areas of Nigeria and Cameroon, but we hope that future validation will take place in wider settings.

## Conclusion

This paper describes the process of co-creating a decision-making framework and quality toolkit for model of care selection in conflict-affected settings. This seems to be a feasible approach for developing tools which are tailored to the context with high participation of stakeholders to use the toolkit. Moreover, the practical application and testing of how to best use the decision-making framework in the process of selecting PHC models of care in conflict settings will be an important next step to allow researchers, practitioners and policymakers to evaluate its actual performance and effectiveness in real-world scenarios.

## Supplementary material

10.1136/bmjgh-2025-019224 online supplemental file 1

## Data Availability

Data sharing not applicable as no datasets generated and/or analysed for this study.
