# Supplementary material for: Co-creating a decision-making framework for primary healthcare models in conflict-affected Cameroon and Nigeria
Source: BMJ Glob Health. 2026 Mar 26;11(3):e019224. doi: 10.1136/bmjgh-2025-019224 (PMC13034324; doi:10.1136/bmjgh-2025-019224)
Supplement: online supplemental file 1 [file bmjgh-11-3-s001.docx]

**A DECISION-MAKING FRAMEWORK AND QUALITY OF CARE TOOLKIT FOR MODELS OF CARE USED AT PRIMARY HEALTH CARE IN CONFLICT-AFFECTED SETTINGS OF CAMEROON AND NIGERIA**

***Version 1***

***July 2023***

**Acknowledgement**

We thank the Ms Lundi-Anne Omam for her dedication and hard work in leading the process of developing this decision-making framework and quality toolkit. We appreciate the technical inputs and support of all the members of the research consortium, led by Dr Rosalind Parkes-Ratanshi and Dr Nicolas Tengdonfor. The research consortium members were: Dr Zara Wudiri, Dr Metuge Alain, Ms Omam Esther, and Mr Mohammed Hassan Ngubdo.

We thank the Health Cluster and Health Sector leads of North West and South West regions of Cameroon (Dr Mustapha Aliyou) and North East Nigeria (Dr Simon Manuel, Dr Yenyi Samuel, Dr Ningi Nuhu Barau, and Beatrice Muraguri) for their great collaboration during the research and in the organisation of the workshops that led to the development of this document.

We extend our heartfelt gratitude to all those who have contributed to the development of this primary health care model of care decision-making framework and toolkit, aimed at providing a comprehensive guide for humanitarian organizations engaged in delivering primary health care in conflict-affected settings of Cameroon and Nigeria

We also extend our sincere gratitude to the research participants of the studies that informed the development of this decision-making framework. We thank the research participants for their invaluable contributions to this vital undertaking. Your willingness to share experiences, insights, and perspectives has been instrumental in shaping the content and recommendations of this primary health care model of care decision-making framework and quality toolkit.

**Purpose and approach**

**Purpose**

This primary health care model of care decision-making framework provides a structured compendium of tools for humanitarian organisations to use to guide the selection and use of models of care to deliver primary health care in conflict-affected settings of Cameroon and Nigeria. This model of care decision-making framework and toolkit will include a pragmatic set of quality considerations across WHO’s seven quality domains.

**The Approach**

This workbook is based on evidence generated through a systematic review, mapping review, a cross sectional survey and qualitative study conducted with the Ministry of health, humanitarian organisations, and conflict affected populations.

This workbook is anchored on the Global Health Cluster Quality of Care in Humanitarian Settings guidelines and WHO’s quality of care in fragile, conflict-affected publication include 07 domains of quality.

From the literature we reviewed, results obtained from the survey and qualitative study coupled to the consultations we had during two workshops of three days each in Cameroon and Nigeria, a collaborative and enhanced curated set of considerations for models of care use in conflict-settings will be arrived at.

Through this co-creation process, humanitarian program designers may be able to work towards improving serve delivery and quality of care for conflict affected populations using different models of care.

This framework and toolkit acknowledges the rapidly changing dynamic of the conflict and environment in which humanitarian organisations operate and provides guidance for delivering PHC in urban, peri-urban, rural and hard-to-reach conflict affected settings.

**Acronyms**

CHW Community Health Worker

NSAG Non-State Armed Group

SOP Standard operating environment

WASH Water Sanitation and Hygiene

RRM Rapid Response Mechanism

**Advantages and Disadvantages of models of care**

This table outlines various models of care utilized in conflict-affected settings, highlighting their general considerations, advantages, and disadvantages. It provides a comparative perspective to guide program designers in choosing the most appropriate model for specific contexts

Table 1: Models of care, strengthens and weaknesses

| **Model of care** | **General considerations** | **Advantages** | **Disadvantages** |
| --- | --- | --- | --- |
| **Health Facilities (HF)**: *A modality of delivering services offered by health personnel in fixed infrastructures like clinics, health centres and hospitals* | - Established facilities could be made use of - Requires staff deployment. - Distance to facility - Could provide quality services. | - Availability of specialized services and medication - Facilities for hospitalisation are available. - Intensive care available or the possibility to establish one. - Relatively Secure - Standardize settings. - Location is well known by Community. - Reduce stigma and increase hope for better outcome. - Good storage facilities - Availability of integrated health services and medication | - Easily targeted during crisis - Populations might travel long distances to get to the health facility. - In some settings, High costs of services provided during crisis compared to mobile clinics and outreach. - Passive follow up of patients. - Health facility can be closed at pick insecurity. - Risk of nosocomial infections - Might have long waiting time. - Health facility can be closed up at pick insecurity. - Risk of nosocomial infections - Might encounter difficulty in referral services due to insecurity. - Frequent staff movement - Many facilities are understaffed health facilities |
| **Mobile clinics (MC):** *Ambulatory approaches of providing preventive and curative health services on an intermittent base, operated by health personnel* | - Complementary approach - Expensive - Hit-and-run - Improves access to services - Provides specialised services - Runs on an established frequency | - Capable of reaching out to a large population within a short space of time. - High yields in terms of health education and sensitization. - Can address multiple health care simultaneously. - Opportunity and access specialized health care. - Transfer of expertise to local actors | - Expensive for the funders - It reduces the use of existing health facilities. - Its only offers periodic care to the community (what happen within). - Great security concerns. - Psychological dependence on specialize service. - Limited ability to carry needed materials - Limited scope of services rendered by mobile team - Critical care services cannot be rendered |

| **Model of care** | **General considerations** | **Advantages** | **Disadvantages** |
| --- | --- | --- | --- |
| **Community based-interventions (CBI)**:  *An approach that uses community health workers to deliver health services. Community health workers is an umbrella term used to refer to community members who are selected by their communities, receive low levels of formal education to provide healthcare in the communities where they live.* | - Services provided by trained community members mostly low skilled - Community members mostly nominate a person they can trust to deliver such services - Will require trainings and close supervisions | - Easy access to health services by the beneficiaries. - Easy to build trust and acceptance through community engagement because human resource is drawn from the community. - Sound knowledge on community health needs. - Ensures effective planning and avoiding misplaced priorities for resources. - Ensures effective planning and avoiding misplaced priorities for resources. - Prevention and promotion health services are more impactful. - Sustainability of community health is guaranteed. - Early detection of diseases of epidemic potential such as Measles, Polio. MonkeyPox etc. - The type of intervention defines the type of CHWs requires | - Inadequate expertise in health service delivery. - There is likely going to be bias in the provision of health services to beneficiaries. - Remoteness of some of the communities makes referral difficult. - Possibilities of some CHWs to go above their job descriptions. - In some cases, confidentiality is a problem. - Unskilled CHWs (Volunteers) - Might face challenges with referral system. - Communities without CHWs - Face record keeping issues. - Issue of documentation and transmission of data - In some cases, confidentiality is a problem. |
| **Home visits (HV):** *This is a modality of healthcare delivery in which the services are provided in the homes of the patients. Although home visits are carried out in community settings, it is a distinct modality of its own as it can be provided by both skilled and non-skilled health workers.* | - No one is missed out - Stigma in home visits is a call for concern especially in the avenue of HIV - Ensure service providers are well trained on how to conduct home visits | - Convenience for delivery of health services - Increased reduction of stigma e.g STIs - The patient or beneficiary benefits - Early detection of diseases of epidemic potential - Easy tracing of defaulters especially for infectious diseases - Easy detection of misuse of supplements - Effectively use in contact tracing and monitoring of contacts - Use in the triage of contacts or cases eg COVID-19 - Easy use to obtain consent and assent in the community | - A costly model of care because there is increase in human resource needed to move to homes, extension of project dateline. - Limit to what the service provider may need to collect as information or carry as working material. - The fact that the services can be provided an unskilled worker is a problem. |
| **Outreach (O)**  *This a modality of service delivery referring to services delivered as an advance strategy of health service from an existing fixed health facility or away from the location where they usually work. This could either be provided by health facilities, mobile clinics, community health workers, private companies, non-governmental organisations, and Ministry of Health* | - Improves access to health care - Improves referrals. | - Affordable for patient - Inclusive (People living with disabilities (PLWD) - Can complement other models to provide a holistic model of PHC delivery - Increase a trust between community, health facility and health service providers - Good for the provision of Psychosocial support (PSS). - Accessible to community - Facilitate referrals to health facility - Allows health facility to get overview of issues of public health concern like outbreaks, WASH, and others. - Strengthens existing health facilities and health systems | - Expensive for service providers - Entails complex logistics - Limited package of services could be delivered - Timing determined by programs, seasons etc - Service delivery determined by providers, not population - Limited people reached due to location and logistics - Does not provide specialist or complex care. - Drain on health facility staff - Easily influenced by other factors like security, bad roads, weather. |

| **Model of care** | **General considerations** | **Advantages** | **Disadvantages** |
| --- | --- | --- | --- |
| **Telemedicine(TM):** *This is a modality of healthcare delivery using mobile applications, telephone calls, text messages or web-based applications* | - Requires access to communication network - Could be done using phone calls, text messages, mobile apps etc | - Fast and bridge the 3Ds delay deciding, delay in the road, delay in the waiting time. - Easy referrals. - Confidentiality and privacy is assured. - Quick and easy access to patient’s health history. - It reduces emergencies because you can capture disease before it gets worst. - Great psychological benefits. | - Poor network connectivity. - Highly dependent on tech. - Lack of physical human touch. - Before referrals you give first aid which will help the patient and prevent death - Client information protection concerns |
| **Hybrid (HY)**   - *This is a modality of delivery health care using at least two models of care* | - Holistic approach - Sustainable - Combines models to provide a synergistic effect | - Better coverage of types of services provided, geographical area and population covered - Better quality of care (access to facility and referral services, improve treatment outcome) - Ensures continuity of care (facility – home/community- facility) - Improves linkage/collaboration - Many more services can be included to improve adherence - Improves community engagement and participation as well - sustainability - Flexibility of delivery model - Better utilization of available resources (human, material and financial); long term cost effective | - Costly to implement - Requires high technical expertise - High operational burden – management challenges - Slow to implement - Easier target (in terms of security with limited insurance package) - Non-utilization of some routine services. |
| **Rapid response mechanisms**  A model to reach inaccessible community during special need | - Transportation is usually by air - Improve access people in special needs - It should be initiated within 72 hours | - It serves a class of people under special needs - It improves access to services - Reduces the course of transfer of cases - It facilitates referral services - It is a holistic approach combining many interventions - Capacitation of local community - It reaches the most inaccessible places | - It is very expensive - Time consuming - It is very short time |

**DECISION MAKING CHECKLISTS**

**Checklist for model of care selection**

The tables below provides a structured way of reflecting on which model of care might be suitable after a certain number of considerations. These are not meant to be prescriptive, but rather highlight a thoughtful process of deciding which model might be preferable under what circumstances.

Table 9‑3: Internal Consideration Questions to guide towards the selection of the most appropriate model of care in humanitarian settings.

| **Considerations** | **Response** | **Recommendation** | **Notes** | **Model under**  **consideration** |
| --- | --- | --- | --- | --- |
| **Barriers** | | | |  |
| Are there frequent ghost towns/lockdowns? | Yes | Consider using Health Facilities | This might depend on the restrictions surrounding the lockdown | Example: Health Facility |
|  | Somewhat | Consider using any Community Health Workers (CHWs) |  |  |
|  | No | Any model of choice |  |  |
| Will patients have to walk for more than one hour to access health care? | Yes | Consider using CHWs |  | -Outreach  -CHW |
|  | Somewhat | Consider using outreach or mobile clinics |  |  |
|  | No | Consider using Health Facility | This might depend on insecurity levels and how much access the organization has |  |
| Will health workers always be available to provide services in the chosen community | Yes | Consider using Health Facility or mobile clinics |  |  |
|  | Somewhat | Consider using Health Facility or mobile clinics or outreach |  |  |
|  | No | Consider using CHWs |  |  |
| Will the community and staff be safe during service delivery? | Yes | Any model of choice |  |  |
|  | Somewhat | Consider using mobile clinics |  |  |
|  | No | Consider using CHWs and outreach |  |  |
| Will the community be accessible all year round? | Yes | Any model of choice |  |  |
|  | Somewhat | Any model of choice |  |  |
|  | No | Consider using CHWs and outreach or mobile clinic |  |  |
| Are there any challenges with regards to communication network? | Yes | Avoid telemedicine, consider any other model |  |  |
|  | Somewhat | Any model of choice |  |  |
|  | No | Any model of choice |  |  |
| Are there specific geographical barriers in the proposed region? | Yes | Consider using CHWs, Telemedicine |  |  |
|  | Somewhat |  |  |  |
|  | No | Consider using Health Facility, Mobile Clinics, Outreaches |  |  |
| Does the Health Facility have the logistical capacity to undertake outreaches? | Yes | Consider using Health Facility, outreach |  |  |
|  | Somewhat | Consider using Mobile Clinics, Community health workers, Health Facilities |  |  |
|  | No | Consider using Mobile Clinics, Community health workers, Health Facilities | Consider supporting outreach logistical support from the project |  |
| Is the community assessable by any means of transportation | Yes | Use any model of choice |  |  |
|  | Somewhat | Accessible by road use Health Facility/Mobile Clinics/Out Reach. If only accessible by air use Rapid Response Mechanism (RRM) |  |  |
|  | No | Telemedicine |  |  |
| Are there newly accessible areas | Yes | Consider using Rapid Response Mechanism (RRM) | This might depend on the safety of the newly accessible areas |  |
|  | Somewhat | Consider using mobile clinics |  |  |
|  | No | Consider using any other appropriate model |  |  |
| **Communities** | | | |  |
| What model do your communities feel is best for them? | Mobile clinic/ Health facility/ Outreach/ Home visit/ Telemedicine/ Hybrid |  |  |  |
| Was the community involved in the planning or need assessment? | Yes | Consider using mobile clinics, home visits, outreach, and Community base invention. |  |  |
|  | No | The community should be considered in all stages of the project |  |  |
|  | Somewhat | Consider using mobile clinics, home visits, outreach, and Community base invention. |  |  |
| Is there a pool of trained CHW in the proposed region or a training plan for the region in place? | Yes | Consider using CHWs |  |  |
|  | Somewhat | Consider using CHWs and another approach | - Also consider consulting with Ministry of Health and communities |  |
|  | No | Consider using Non CHW model |  |  |
| Does the communication network allow for telephone or internet calls? | Yes | Consider using Telemedicine |  |  |
|  | Somewhat | Consider using telemedicine and other approach |  |  |
|  | No | Consider using non-Telemedicine models |  |  |
| Is the health seeking behaviour in the locality in favour of the use of modern medicine? | Yes | Consider using Health Facility, outreach, Telemedicine |  |  |
|  | Somewhat | Consider using Mobile Clinics, Community Health Workers |  |  |
|  | No | Consider using Mobile Clinics, Community Health Workers |  |  |
| **Health Financing** | | | |  |
| Are communities capable of paying for services? | Yes | Consider Health facility, Mobile Clinic | Consider introducing vouchers |  |
|  | Somewhat | Health facility | Some services will be paid for, while some will not be paid for. |  |
|  | No | Consider Mobile clinics, outreach programs | Considering that we are working in a humanitarian setting, all services will be free of charge. |  |
| Can the population targeted afford to pay for transport to reach the HF? | Yes | Consider health facility |  |  |
|  | Somewhat | Consider Mobile Clinic and Outreach |  |  |
|  | No | CHW (mobile or outreach) | Consider Telemedicine if can be provided in a cheap manner on simple phones |  |
| Can the population targeted afford to pay for care at the HF? | Yes | Consider Heal Facility |  |  |
|  | Somewhat | Consider CHW, outreach | If Health Facility are free or if vouchers or direct payment are available (by yourself or other organizations) |  |
|  | No | Consider Mobile Clinic, CHW | If Health Facility are free or if vouchers or direct payment are available (by yourself or other organizations). Consider Telemedicine if can be provided in a cheap manner on simple phones |  |
| Is there a functional HF within reachable distance (safe to reach, affordable to reach and within one hour walk)? | Yes | Consider health facility |  |  |
|  | Somewhat | Consider Outreach, CHW |  |  |
|  | No | Consider Outreach, Mobile Clinics, CHW | Consider Telemedicine if can be provided in a cheap manner on simple phones or there is good internet in the area and if a health worker is nearby.  Can the project make the Health Facility functional? E.g train & maintain staff, add utilities, repair infrastructure, equipment – if yes, consider Health Facility) |  |
| Is there adequate financial resources to support activities | Yes | Consider using the most appropriate and acceptable model |  |  |
|  | Somewhat | Consider using community-based model | Consult with communities and other partners |  |
|  | No | Consider using community-based model |  |  |
| **Decision making priority**  **(Total the number of times each model was considered)** | | **Total Health Facility =**  **Total Outreach =**  **Total CHWs =**  **Total Home Visits =**  **Total Mobile clinic =**  **Total Telemedicine =**  **Rapid Response Mechanism =**  **Ambulances =**  **Total Hybrid (more than one model) =** | | |

Table 9‑4: Internal Consideration Questions to guide towards the selection of the most appropriate model of care in humanitarian settings.

| **Considerations** | **Response** | **Recommendation** | **Notes** | **Model under**  **consideration** |
| --- | --- | --- | --- | --- |
| **Organizational capacity** | | |  |  |
| Which of the models does your organization have skill personnel for? | Mobile clinic/ Health facility/ Outreach/ Home visit/ Telemedicine/ Hybrid | Consider using ant model(s) with skill personnel | - Consider re-fresher trainings for staff - Consider trainings and recruiting skilled human resources - Consider using a feasible model |  |
| Does your organization have experience with supportive supervision for CHWs? | Yes | Consider using CHWs | - Must ensure provision of commodities for CHWs |  |
|  | Somewhat | Consider using CHWs | - Ensure staff are trained to work with and support CHWs |  |
|  | No | Consider not using CHWs |  |  |
| Is there a staff evacuation/mitigation plan in place by cluster co-ordination or stakeholders? | Yes | Consider Health Facility, Outreach | - As long as communities can get to the facility |  |
|  | Somewhat | Consider Health Facility |  |  |
|  | No | Consider Mobile Clinic, CHW, Telemedicine |  |  |
| Will the services provided require skilled health care? | Yes | Consider using Health Facility or mobile clinics |  |  |
|  | Somewhat | Consider using outreach |  |  |
|  | No | Consider using community-based model |  |  |
| Does the organization have the logistical capacity to undertake MC? | Yes | Consider using Mobile Clinics |  |  |
|  | Somewhat | Consider using non-mobile Clinic models | Only consider using mobile clinics if the organisations capacity will be strengthen or supported by the donor, another organisation or MOH |  |
|  | No | Consider using Non-mobile Clinic models |  |  |
| Do we have the capacity to provide services using Telemedicine? | Yes | Consider using Telemedicine |  |  |
|  | Somewhat |  |  |  |
|  | No | Consider not using Telemedicine |  |  |
| Is there a restriction from partners entering or leaving the proposed locality? | Yes | Consider using Community based interventions, Telemedicine | Use of military medics/vigilante groups could be considered dependent on local context. |  |
|  | Somewhat | Consider using Health Facility, outreach | Also consider access negotiations to ensure staff and patients safety |  |
|  | No | Consider using Health Facility, outreach |  |  |
| Are there other reasons from the community impeding the use if health facilities (e.g. stigma, insecurity, lack of confidence, culture)? | Yes | Consider using Mobile clinics, Community based interventions or Telemedicine | Consider outreach if appropriate |  |
|  | Somewhat | Consider using health facility, outreaches | Also consider using Mobile clinics or Community based interventions or Telemedicine |  |
|  | No | Consider using health facility, outreaches |  |  |
| Will your proposed intervention or situation require specialized services (e.g. high trauma levels)? | Yes | Consider using Health facilities, outreaches, or mobile clinics | Avoid CHWs |  |
|  | Somewhat | Consider using Health facilities, outreaches, or mobile clinics | Consider using CHWs to support referrals |  |
|  | No | Consider using community based interventions, Telemedicine |  |  |
| Do you need to provide a wide range of services? | Yes | HF, outreaches | Outreaches need strong referral mechanisms |  |
|  | Somewhat |  |  |  |
|  | No | Other models |  |  |
| **Decision making priority**  **(Total the number of times each model was considered)** | | **Total Health Facility =**  **Total Outreach =**  **Total CHWs =**  **Total Home Visits =**  **Total Mobile clinic =**  **Total Telemedicine =**  **Rapid Response Mechanism =**  **Ambulances =**  **Total Hybrid (more than one model) =** | | |

**General Considerations**

**Table 3:** General considerations which apply to all models of care but are relevant and should be considered strongly in humanitarian health programming

| **Considerations** | **Response** | **Recommendation** |
| --- | --- | --- |
| **COMMUNITIES** | | |
| Will beneficiaries/ patients be required to pay for health care at the point of care | Yes | Consider collaborating with any other implementing partner whose program covers financing aspects of health care |
|  | Somewhat | Consider using voucher system to facilitate financing at point of care |
|  | No | Consider using voucher system to facilitate financing at point of care |
| Will beneficiaries/ patients be required to pay for health care at the point of care | Yes | Consider collaborating with any other implementing partner whose program covers financing aspects of health care |
|  | Somewhat | Consider using voucher system to facilitate financing at point of care |
|  | No | Consider using voucher system to facilitate financing at point of care |
| Did communities contribute to the selection of a model of care that best meets their needs? | Yes | Consider using any of the models chosen by communities |
|  | Somewhat | Consider engaging communities in the selection of a model which best meets their needs |
|  | No | Consider engaging communities in the selection of a model which best meets their needs |
| Was the community involved in the planning or need assessment? | Yes | Consider using mobile clinics, home visits, outreach and Community base invention. |
|  | No | The community should be considered in all stages of the project |
|  | Somewhat | Consider using mobile clinics, home visits, outreach and Community base invention. |
| Are the services provided based on the need of the community? | Yes | Consider engaging the community in the selection of their needs in order not to waste resources |
|  | Somewhat |  |
|  | No |  |
| Will the community members be recruited as services providers? | Yes | Consider community members of good integrity. |
|  | Somewhat | Select some community members, train them and recruit them for the sustainability of the project. |
|  | No |  |
| Does your intervention factor PWD and gender in their activities? | Yes | Consider gender and disability inclusion in all activities. |
|  | No |  |
|  | Somewhat | Develop a gender and disability safeguarding policies. |
| Are there community structures and will there be available for support, planning, implementation and evaluation. | Yes | Factor the accountability to affected population. |
|  | Somewhat |  |
|  | No |  |
| Was the community involved in the planning or need assessment? | Yes | Consider using mobile clinics, home visits, outreach and Community base invention. |
|  | Somewhat | Consider using mobile clinics, home visits, outreach and Community base invention. |
|  | No | The community should be considered in all stages of the project |
| Are the services provided based on the need of the community? | Yes | Consider engaging the community in the selection of their needs in order not to waste resources |
|  | Somewhat |  |
|  | No |  |
| **FUNDING AND ORGANIZATION CAPACITY** | | |
| Can the model of care be sustained beyond funding period? | Yes | Consider using any model of care which could be sustainable after funding ends |
|  | Somewhat | Consider engaging with Ministry of Health and communities to select a model which could be sustained by them |
|  | No | Consider engaging with Ministry of Health and communities to select a model which could be sustained by them |
| Does your organization have experience delivering PHC in conflict settings? | Yes | Consider developing standard operating procedures for your operations for standardization |
|  | Somewhat |  |
|  | No |  |
| Does your organization have a sustainability plan? | Yes |  |
|  | Somewhat | Consider engaging other sources of income such as cost sharing, and user fees |
|  | No |  |
| Does your organization have experience with supportive supervision for CHWs? | Yes | Consider working with Community health workers (CHWs) |
|  | Somewhat |  |
|  | No | Consider getting trained on working with CHWS |
| How does your proposed model of care contribute to strengthening the health system? | Yes | Consider revisiting the health sector response plan and the national health strategy for the country |
|  | Somewhat |  |
|  | No |  |
| How can you make your model as sustainable as possible? | Yes | Consider engaging with community members and other stakeholders to develop a sustainability plan/ strategy |
|  | Somewhat |  |
|  | No |  |
| Completely inaccessible due to security challenges | Yes | Consider using Rapid response mechanism |
|  | Somewhat | Consider using the Mobile clinic mobile/ outreach |
|  | No | Select other most appropriate models |
| **COORDINATION** | | |
| Is there an established functional coordination system/mechanism at local level | Yes | Consider joining the coordination system and share updates of activities |
|  | Somewhat | Consider joining to contribute in strengthening the coordination system |
|  | No | Consider advocating for one to be establish through OCHA, WHO or any other partners on the ground |
| Is there a proper logistics management and coordination system in place | Yes | Consider a combination of models |
|  | Somewhat |  |
|  | No | Consider using Mobile outreach |

**Toolkit for assessing Quality of care for primary health care services delivered at conflict-affected settings**

1. **Quality of care assessment tool**

This toolkit was developed to guide program designers in humanitarian organizations and Ministry of Health to assess and reflect on quality-of-care considerations for their programs prior program implementation (before a particular model of care has been chosen to delivery primary health care services)

**Table 1: Quality of care assessment tool (Prior to program implementation)**

|  | **Yes/NO** | **Notes** |
| --- | --- | --- |
| **EFFECTIVE** | | |
| Does the organization have appropriate guidelines and SOPs |  |  |
| Have all staff been trained on appropriate SOPs |  |  |
| Does your SOPs align or respect national guidelines |  |  |
| Will you conduct poet patients/clients satisfaction of services received? |  |  |
| **EFFICIENT** | | |
| Are there local skilled human resources whose capacities could be strengthened to deliver health services to minimize cost of recruiting external expertise? |  | Human resource as a whole not just for health |
| Are there existing models of care whose capacities could be strengthened to minimize cost of implementing a new model of care? |  | Existing models of care |
| Will it be challenging to mobilizing additional resources to sustain your interventions |  | If yes, consider alternative partnerships and sources of funding |
| Is the model of care used to deliver services sustainable? |  |  |
| Do you have sufficient logistics in place to support your services? |  |  |
| Was an assessment of the needs and priorities from communities conducted to inform interventions |  |  |
| Are there existing health facilities whose capacities could be strengthened to minimize cost of implementing a new model of care? |  |  |
| Does your organization have a sustainability or exit plan |  |  |
| Will there be need for equipment need to be transported during service delivery? |  |  |
| **INTEGRATED** | | |
| Is there a clear two-way Referral pathway to ensure appropriate and complete referrals? |  | Sectors within their coordination should establish clear referral pathways between; sector-sector, within the organization, government-government, government-partner, and partner-partner. |
| Will an organization or ministry of health cover associated cost of referrals and health care of the patient being referred? |  | Patients with chronic disease and non-communicable diseases are usually excluded from humanitarian interventions.  If no, how will patients be required to pay? |
| Will Helicopter be required to facilitate referrals of patients? |  | The situation of the patient/emergency associated with disease condition and availability of resources. |
| Will a motor bike be required to facilitate referrals? |  | The situation of the patient/emergency associated with disease condition and availability of resources. |
| Will a bus or car/ambulance be required to facilitate referrals? |  | The situation of the patient/emergency associated with disease condition and availability of resources. |
| Will canoe/boats be required to facilitate referrals? |  |  |
| Is your organization a member/involved in the Coordination system or cluster, or any working group in place? |  | Is there a coordination system in place (Sub National and National)? |
| Has a coordination mechanism with beneficiary communities been establish? |  | Could serve as feedback mechanism for programs and improve accountability of interventions |
| Has a coordination mechanism with government been established |  | if yes, consider accessing it to improve approval rates of projects |
| Are there other coordination mechanism between similar organizations? |  | Is yes, be a member, if no, encourage similar ones to have one |
| Has an exit plan been developed? |  | If no, please develop one |
| Are there other organizations implementing same intervention in the same locality? |  | If yes, please target a different locality |
| Are there other departments in your institution implementing cross cutting interventions? |  | If yes, ensure smooth coordination |
| Is there a mechanism in place to jointly implement multisectoral interventions within the organization? |  |  |
| Does your programming capture and attend to cross cutting issues (protection, GBV, PSEA, AAP, PLWD) in your interventions? |  | If no, mainstream these |
| Are there resources to ensure interventions are delivered using an integrated lens? |  |  |
| Are there partners in different sectors with whom these interventions could be jointly delivered? |  |  |
| With local authorities |  |  |
| Does your organization have clear SOPs for services and interventions to be implemented? |  | Clear SOPs is important to ensure focused services |
| Do you have a map of partners intervening in the communities you are targeting? |  | Partners mapping ensure proper synergy among actors |
| **PATIENT CENTERED** | | |
| Are the Donor preferences patient centered? |  |  |
| Are these services gender sensitive? |  |  |
| Is there a hot line that patients can call during emergencies? |  |  |
| Have you held planning meetings with communities? |  |  |
| Are your interventions designed to serve your individuals in your target communities? |  |  |
| Are services delivered based on needs of groups/individuals in the communities? |  |  |
| Are there clearly defined beneficiary criteria? |  |  |
| Is there an organization policy that guides the attitudes and behavior of service providers towards beneficiaries? |  |  |
| Are referral systems active (service providers accompany beneficiaries and provide services on cross cutting issues) |  |  |
| Are beneficiaries aware of the project package (including duration, services, inclusion criteria)? |  |  |
| Is there a mechanism in place for accountability, feedback and complaint by the affected population? |  |  |
| Will patient information, education and communication be part of services to be delivered? |  |  |
| **SAFETY** | | |
| Is the targeted community reachable? |  | Highlight |
| Do you have a risk assessment and safety plan? |  | This includes risks associated with commodities, incidences etc |
| Have the safety concerns within the project been identified and appropriately addressed? |  | Specify and plan mitigations in your risk assessment plan |
| Have there been a previous attack in the community? |  |  |
| Will key community gate keepers present? |  |  |
| Do gate keepers still have influence in their community? |  |  |
| Is there an access negotiation plan for delivery of services and commodities? |  |  |
| Are there dedicated personnel within the organization or project in charge of safety? |  |  |
|  |  |  |
| Is your organization perceived as being neutral by the public and other stakeholders? |  |  |
| Have the administrative authorities approved of your interventions? |  |  |
| Inform administration  Are administrative authorities at all level informed of your interventions? |  |  |
| **TIMELY** | | |
| Do you have qualified and sufficient number of staff to deliver the services? |  |  |
| Are there resources available to provide the needed services at all times? |  |  |
| Can beneficiaries easily access services within one hour walkable distance? |  |  |
| Are night emergency services available to beneficiaries at all times? |  |  |
| Is there preposition emergency |  |  |
| **EQUITABLE a fair and impartial system that insures access to care to all groups** | | |
| Are your interventions designed to serve your individuals in your target communities? |  |  |
| Are services delivered based on needs of groups/individuals in the communities? |  |  |
| Are there clearly defined beneficiary criteria? |  |  |
| Are service providers receptive toward beneficiary? |  |  |
| Are referral systems active (service providers accompany beneficiaries and provide services on cross cutting issues) |  |  |
| Are beneficiaries aware of the project package (including duration, services, inclusion criteria)? |  |  |
| Is there a mechanism in place for accountability, feedback and complaint by the affected population? |  |  |
| Are there considerations for the vulnerable and less privilege in the provision of their services? |  | Vulnerable groups, Disable, elderly, women and children etc… |
| Do you have gender balance work force? |  |  |
| Have all staff receive training of safeguarding? |  |  |

1. **Quality toolkit per model of care**

This toolkit was developed to guide quality reflections and considerations for each chosen model of care.

Table: Quality of care evaluation tool (during implementation)

|  | **Health Facilities** | **Community Based interventions** | **Mobile Clinic** | **Outreach** | **Home visits** | **Telemedicine** |
| --- | --- | --- | --- | --- | --- | --- |
| **Safety**  *Avoid harm to people for whom care is intended* | Is the health facility close to a security post/safe location?  Is there provision for satellite phones or two-way radio communication?  Is the health facility fenced?  Does the health facility have a trained security personnel and means of communication?  Is there an incidence reporting system in place?  Are there effective infection control measures at the level of the health facility (availability of PPE, Hand washing station, proper waste management procedures, etc. ?  Is there an emergency exit plan in case of attack?  Are health workers trained to implement the exit emergency plan?  Does the working plan for staff flexible enough to prevent burnout?  Are there enough recreational facilities for staff for work life balance.    Are there provision in place for mental health and psychosocial support for staff in the health facility? | Is there provision for training on safety measures at work place?  Is there a safety plan for CHWs in case of an incidence?  Is there an incidence reporting system in place?  Is there a system in place for sharing safety/security information among CHWs?  Are there effective infection control measures at the level of the health facility (availability of PPE, Hand washing station, proper waste management procedures, etc. ?  Are there provisions in place for mental health and psychosocial support for CHWs? | Are there security personnel to accompany the mobile clinic?  Is there provision for satellite phones or two-way radio communication?  Are field movement informed by security advisory?  Is there provision for bullet proof jacket for the outreach team?  Is there any backup equipment to ensure functionality of the mobile clinic?  Is the safety of the patients taken into consideration when selecting the service delivery point?  Are there effective infection control measures at the level of the health facility (availability of PPE, Hand washing station, proper waste management procedures, etc?  Is there an emergency exit plan in case of attack?  Are health workers trained to implement the exit emergency plan?  Does the working plan for staff flexible enough to prevent burnout?  Are there enough recreational facilities for staff for work life balance.    Are there provision in place for mental health and psychosocial support for staff in the mobile team?  Is there an incidence reporting system in place?  Do they have means of communication in times of attacks or danger? | Are field movement informed by security advisory?  Is there provision for satellite phones or two-way radio communication?  Is there provision for bullet proof jacket for the outreach team?  Is the safety of the patients taken into consideration when selecting the service delivery point?  Are there effective infection control measures at the level of the health facility (availability of PPE, Hand washing station, proper waste management procedures, etc. ?  Is there an emergency exit plan in case of attack?  Are health workers trained to implement the exit emergency plan?  Does the working plan for staff flexible enough to prevent burnout?  Are there enough recreational facilities for staff for work life balance.    Are there provision in place for mental health and psychosocial support for staff in the mobile team?  Is there an incidence reporting system in place?  How often do they meet with the community to discuss security issues?  Is there any agreement between the outreach team and the community before proceeding on a particular service to be rendered? | Is consent obtained from community leaders and heads of various family before going out to visit?  Is there an incidence reporting system in place?  Are there trained on home visit protocol?  Is there any advocacy paid to the ward head on the purpose of the home visit? | Is there provision for satellite phones or two-way radio communication?  Is there a good system in place for client personal and data protection?  Is there any maintenance of the communication mechanism in place?  (move to effectiveness) |
| **Patient Centered**  *Providing care that responds to individuals preferences, needs and values* | Is there any quick referral pathway for patient to access other medical services when needed?  Are the patients educated on the services available and the choice they have?  Are service provision units clearly labelled for easy navigation?  Is there an available patient feedback mechanism at the health facility (suggestion boxes, toll-free line, etc…?  Is the feedback from the patient considered in planning of care?  Is care provided based on informed choices by the patient?  Are there available IEC materials to help the patient to access a particular service?  Will cultural considerations been taken into account in the delivery of care?  Is the patient consent sought before delivery of care?  Are beneficiary aware of the service package, working hours and days of the health facility?  Is the service provided to PLWD and other vulnerable /marginalized groups friendly and free of stigma? | Are the patients educated on the services available and the choice they have?  Is there an available patient feedback mechanism at the community level (suggestion boxes, toll-free line, etc…?  Is the feedback from the patient considered in planning of care?  Is care provided based on informed choices by the patient?  Are there available IEC materials to help the patient to access a particular service?  Will cultural considerations be taken into account in the delivery of care?  Are beneficiary aware of the service package, working hours and days of the CHWs?  Is the service provided to PLWD and other vulnerable /marginalized groups friendly and free of stigma? | Are the patients educated on the services available and the choice they have?  Are service provision units clearly labelled for easy navigation?  Is there an available patient feedback mechanism (suggestion boxes, toll-free line, etc…?  Is the feedback from the patient considered in planning of care?  Is care provided based on informed choices by the patient?  Are there available IEC materials to help the patient to access a particular service?  Will cultural considerations be taken into account in the delivery of care?  Is the patient consent sought before delivery of care?  Are beneficiary aware of the service package, working hours and days of the mobile clinic?  Is the service provided to PLWD and other vulnerable /marginalized groups friendly and free of stigma?  Is there a linkage to the nearest functional health facility for continuation of care for patient seen by the mobile clinic? | Are the patients educated on the services available and the choice they have?  Are service provision units clearly labelled for easy navigation?  Is there an available patient feedback mechanism (suggestion boxes, toll-free line, etc…?  Is the feedback from the patient considered in planning of care?  Is care provided based on informed choices by the patient?  Are there available IEC materials to help the patient to access a particular service?  Will cultural considerations be taken into account in the delivery of care?  Is the patient consent sought before delivery of care?  Are beneficiary aware of the service package, working hours and days of the outreach team?  Is the service provided to PLWD and other vulnerable /marginalized groups friendly and free of stigma?  Is there a linkage to the nearest functional health facility for continuation of care for patient seen by the outreach team?  Are the community involved in the decision making before the outreach visit?  Is there a good IEC system to inform the patients before the outreach activity | Are the patients educated on the services available and the choice they have?  Is there an available patient feedback mechanism at the community level ( toll-free line, etc…?  Is the feedback from the patient considered in planning of care?  Is care provided based on informed choices by the patient?  Are there available IEC materials to help the patient to access a particular service?  Will cultural considerations be taken into account in the delivery of care?  Are beneficiary aware of the service package, working hours and days of the CHWs?  Is the service provided to PLWD and other vulnerable /marginalized groups friendly and free of stigma? | Are the community properly sensitized for the use of telemedicine on healthcare delivery?  Do patients or careers have mobile gadgets?  Are beneficiary aware of the service package, working hours and days of the health facility?  Is the service provided to PLWD and other vulnerable /marginalized groups friendly and free of stigma? |
| **Efficient**  *Maximizing the benefit of available resources and avoid waste* | Are there adequate human resources for health?  Is there existing and functional equipment for service delivery?  Does the facility have sufficient storage for commodities?  Is there effective logistic management in place for supplies? | Are there available local skilled workers resident within the community?  Have resources that will be needed at the community level been identified?  Is there a logistics system for the movement of commodities? | Have modalities that will be used to run the mobile clinic been agreed upon?  Have services that will be provided and referral pathways identified?  Are there sufficient logistics arrangements to support mobile services? | Have modalities for the outreach services been agreed upon?  Have services that will be provided and referral pathways identified?  Are there sufficient logistics arrangements to support outreach services? | Are there adequate skilled personnel to conduct home visits in the community?  Are there adequate referral pathways identified?  Are there available/ adequate resources to support community health volunteers? | Is there effective network coverage in the affected communities?  Is there sufficient skilled medical personnel to provide services in the major/ multiple languages of the affected population?  Are there adequate platforms and communication equipment? |
| **Effective**  *Providing evidence-based health care services to those who need them* | Are there guidelines/SOPs in the health facility for the delivery of services?  Have health workers been trained on those guidelines and SOPs?  Have health facility staff been trained on stock management?  Have key performance indicators been set? | Are there guidelines/SOPs in place for community-based interventions?  Have CHWs/CVs been trained on those guidelines and SOPs?  Are CHWs/CVs trained on data management/record keeping and reporting? (cut across)  Are mobile clinic staff oriented on stock management? | Are there guidelines/SOPs in place for running a mobile clinic?  Have all mobile clinic staff been trained on appropriate guidelines/SOPs?  Are CHWs/CVs trained on data management/record keeping and reporting?  Are mobile clinic staff oriented on stock management? | Are there guidelines/SOPs in place for outreach services?  Have all outreach staff been identified and trained on appropriate guidelines/SOPs?  Are outreach staff oriented on stock management?  Are CHWs/CVs trained on data management/record keeping and reporting? | Are there guidelines/SOPs on how to conduct home visits?  Have the personnel been trained on those guidelines/SOPs?  Are health personnel been trained in record-keeping and reporting?  Are CHWs/CVs trained on data management/record keeping and reporting? | Are there guidelines or SOPs on how to provide telemedicine?  Have the service providers been adequately trained on the guidelines/SOPs?  Is there a clear referral pathway in place?  Are operators trained on data management/record keeping and reporting? |
| **Equitable**  *Providing health care that does not vary according to age, gender, religion etc* | Are health care workers trained on humanitarian and Do No Harm principles?  Are there considerations in place for the provision of gender-sensitive services including indicators?  Are there considerations in place for the provision of people with special needs (PLWDs etc) including indicators?  Are there mechanisms available for complaints and feedback from beneficiaries?  Have all cultural consideration’s been taken into account in the delivery of care | Are health care workers trained on humanitarian and Do No Harm principles?  Are there considerations in place for the provision of gender-sensitive services including indicators?  Are there considerations in place for the provision of people with special needs (PLWDs etc) including indicators?  Are there mechanisms available for complaints and feedback from beneficiaries?  Have all cultural consideration’s been taken into account in the delivery of care | Are health care workers trained on humanitarian and Do No Harm principles?  Are there considerations in place for the provision of gender-sensitive services including indicators?  Are there considerations in place for the provision of people with special needs (PLWDs etc) including indicators?  Are there mechanisms available for complaints and feedback from beneficiaries?  Have all cultural consideration’s been taken into account in the delivery of care | Are health care workers trained on humanitarian and Do No Harm principles?  Are there considerations in place for the provision of gender-sensitive services including indicators?  Are there considerations in place for the provision of people with special needs (PLWDs etc) including indicators?  Are there mechanisms available for complaints and feedback from beneficiaries?  Have all cultural consideration’s been taken into account in the delivery of care | Are health care workers trained on humanitarian and Do No Harm principles?  Are there considerations in place for the provision of gender-sensitive services including indicators?  Are there considerations in place for the provision of people with special needs (PLWDs etc) including indicators?  Are there mechanisms available for complaints and feedback from beneficiaries?  Have all cultural consideration’s been taken into account in the delivery of care | Are health care workers trained on humanitarian and Do No Harm principles?  Are there considerations in place for the provision of gender-sensitive services including indicators?  Are there considerations in place for the provision of people with special needs (PLWDs etc) including indicators?  Are there mechanisms available for complaints and feedback from beneficiaries?  Have all cultural consideration’s been taken into account in the delivery of care |
| **Timely**  *Reducing waiting times and harmful delays for both those who recieve care and those who give care* | Is the estimated patient waiting time within 30 minutes of arrival at the health facility?  Is there an effective ambulance system in place?  Are there designed facility linkages for referrals (counter referral to CHWs and referral for higher level of care)?  Are there adequate medications and supplies for emergencies | Is the estimated response time for a patient within 30 minutes of call?  Are there sufficient number of staff to deliver service for each community based on national guidelines?  Is there a referral system to link patients to health facility?  Does the CHWs respect appointments made with the patients?  Would the CHWs attend to the patients as planned in the session plan | Is the estimated patient waiting time within 30 minutes of arrival at the mobile clinic service delivery point?  Are mobile sessions to be implemented base on the community availability?  Do mobile clinics respect their appointments with the communities?  Are there designed facility linkages for referrals (counter referral to CHWs and referral for higher level of care)?  Are the mobile teams trained on the emergency ambulance system for referral? (under effective) | Is the estimated patient waiting time within 30 minutes of arrival at the service delivery point?  Are outreach sessions to be implemented base on the community availability?  Are there designed facility linkages for referrals (counter referral to CHWs and referral for higher level of care)? | Is there an itinerary for home visit?  Is the duration of home visit at least 30 minutes?  Are there sufficient number of staff to deliver service for home visit?  Is there a referral system to link patients to health facility?  Are there planned follow up home visit? | Is the maximum expected patient waiting time within 30 minutes?  Is there dedicated 24/7 Toll free lines?  Is there multi lingual operator attending the Toll free line? (under equity)  Is there a referral system to link patients to health facility? |
| **Integrated**  *Health care is coordinated across all levels and partners and between sectors* | **Example**:  Number of interventions to be delivered with at least more than one health service to offer  Is there an integrated service delivery package at the health facility based on sphere standards?  Is there an integrated reporting tool in place with clear indicators?  Are there two ways integrated referral system? Replace  Is there a multi-sectoral collaboration in the delivery of services at the health facility? | How long would it take to respond patients for an integrated service?  Are there qualified sufficient number of staff to deliver integrated services  Are session plans developed in an integrated manner (ICCM, basic PSS, NCDs, WASH, Lactation counselling etc)  Are there two ways integrated referral system? | Are there trained skill personnel providing integrated services  Are integrated mobile clinic having readily available essential drugs  Is there an integrated service delivery package at the health facility based on sphere standards?  Is there an integrated reporting tool in place with clear indicators?  Is there coordination with other service providers to promote synergy and avoid duplication?  Are mobile sessions planned in an integrated manner  Are there two ways integrated referral system? | Are there trained skill personnel providing integrated services  Are integrated outreaches having readily available essential drugs  Is there an integrated service delivery package at the health facility based on sphere standards?  Is there an integrated reporting tool in place with clear indicators?  Is there coordination with other service providers to promote synergy and avoid duplication?  Are outreach sessions planned in an integrated manner  Are there two ways integrated referral system? | Are there trained skill personnel providing integrated services  Are there available services to provide integrated home services  Are home visits planned in an integrated manner (ICCM, basic PSS, NCDs, WASH, Lactation counselling etc)  Is there an integrated reporting tool for home visits in place with clear indicators?  Are there two ways integrated referral system? | Is there multi lingual operator attending the Toll free line for provision of integrated services  Is there an integrated reporting tool for home visits in place with clear indicators?  Are there two ways integrated referral system? |

**Contributors**

The following took part in workshop that led to the development of this toolkit.
